# Development of an intervention to increase adherence to nebuliser treatment in adults with cystic fibrosis: CFHealthHub

**DOI:** 10.1186/s40814-020-00739-2

**Published:** 2021-01-04

**Authors:** M. A. Arden, M. Hutchings, P. Whelan, S. J. Drabble, D. Beever, J. M. Bradley, D. Hind, J. Ainsworth, C. Maguire, H. Cantrill, A. O’Cathain, M. Wildman

**Affiliations:** 1 Centre for Behavioural Science and Applied Psychology, Sheffield Hallam University, 2.03a Heart of the Campus, Collegiate Crescent Campus, Sheffield, S10 2BQ UK; 2 Sheffield Adult Cystic Fibrosis Unit Sheffield Teaching Hospitals NHS Foundation Trust, Northern General Hospital, Herries Road, Sheffield, S5 7AU UK; 3 Health eResearch Centre—Farr Institute, Division of Imaging, Informatics and Data Sciences, School of Health Sciences, Faculty of Biology, Medicine and Health, The University of Manchester, Manchester Academic Health Science Centre, Manchester, M13 9GB UK; 4 School of Health and Related Research, University of Sheffield, Regent Court, 30 Regent Street, Sheffield, S1 4DA UK; 5 Clinical Trials Research Unit, University of Sheffield, Regent Court, 30 Regent Street, Sheffield, S1 4DA UK; 6 Centre for Experimental Medicine, School of Medicine, Dentistry and Biomedical Sciences, Queen’s University, 97 Lisburn Road, Belfast, BT9 7BL UK

**Keywords:** Cystic fibrosis, Adherence, Intervention development, Behaviour change wheel, Person-based approach, Digital intervention, Habit formation

## Abstract

**Background:**

Cystic fibrosis (CF) is a life-limiting genetic condition in which daily therapies to maintain lung health are critical, yet treatment adherence is low. Previous interventions to increase adherence have been largely unsuccessful and this is likely due to a lack of focus on behavioural evidence and theory alongside input from people with CF. This intervention is based on a digital platform that collects and displays objective nebuliser adherence data. The purpose of this paper is to identify the specific components of an intervention to increase and maintain adherence to nebuliser treatments in adults with CF with a focus on reducing effort and treatment burden.

**Methods:**

Intervention development was informed by the Behaviour Change Wheel (BCW) and person-based approach (PBA). A multidisciplinary team conducted qualitative research to inform a needs analysis, selected, and refined intervention components and methods of delivery, mapped adherence-related barriers and facilitators, associated intervention functions and behaviour change techniques, and utilised iterative feedback to develop and refine content and processes.

**Results:**

Results indicated that people with CF need to understand their treatment, be able to monitor adherence, have treatment goals and feedback and confidence in their ability to adhere, have a treatment plan to develop habits for treatment, and be able to solve problems around treatment adherence. Behaviour change techniques were selected to address each of these needs and were incorporated into the digital intervention developed iteratively, alongside a manual and training for health professionals. Feedback from people with CF and clinicians helped to refine the intervention which could be tailored to individual patient needs.

**Conclusions:**

The intervention development process is underpinned by a strong theoretical framework and evidence base and was developed by a multidisciplinary team with a range of skills and expertise integrated with substantial input from patients and clinicians. This multifaceted development strategy has ensured that the intervention is usable and acceptable to people with CF and clinicians, providing the best chance of success in supporting people with CF with different needs to increase and maintain their adherence. The intervention is being tested in a randomised controlled trial across 19 UK sites.

**Supplementary Information:**

The online version contains supplementary material available at 10.1186/s40814-020-00739-2.

## Key messages regarding feasibility

- In order to develop an intervention to increase adherence to treatment in people with cystic fibrosis (CF), we needed to develop an intervention using behavioural science theory and evidence and informed by people from our target population.
- We developed and refined a complex intervention underpinned by a strong theoretical framework and evidence base. The CFHealthHub intervention is usable and acceptable to people with CF, providing support for people with CF with different needs to increase and maintain their adherence.
- We have a digital platform, intervention manual, and training package for use in the main trial of the CFHealthHub intervention

## Background

Cystic fibrosis (CF) is an inherited genetic condition that affects approximately 10,500 people in the UK and 100,000 worldwide [1]. The condition causes the build-up of thick sticky mucus in the digestive system and lungs which can result in recurrent lung infections, progressive lung damage, and respiratory failure [2]. People with CF require a time-consuming regimen of treatment in order to maintain their health [3, 4].

There are effective inhaled treatments for CF, usually delivered via a nebuliser, that include antibiotics to reduce infections and mucolytics to thin mucus and to keep airways clear. However, consistent with other long-term conditions, adherence to nebuliser treatments is low [5–7]. Low adherence is associated with increased lung damage and additional need for treatment with intravenous (IV) antibiotics, with higher associated treatment costs [6, 8–11], and significant impacts on quality of life [12]. There is a need for effective interventions to increase adherence to treatment in this population.

Interventions have so far shown limited success in increasing adherence in people with CF [13–15]. There are a number of potential reasons for this. First, the interventions may not be targeting the most appropriate factors [16]. Second, there is a lack of studies using a theory and evidence-based approach [17]. Third, interventions may assume that one-size fits all despite evidence that the factors affecting adherence may be person-specific [18]. Even where there have been reported successes, adherence outcomes have not been measured objectively and therefore the findings may not be reliable. Adherence is often measured by either self-report or medicine possession ratio (MPR). In the UK, objective estimates of median adherence are in the region of 36% [5], whereas MPR for inhaled therapy are in the region of 65% [11] and self-report around 80% [5, 11]. To be sure of success, we need to be able to assess the impact of an intervention on sensitive, objectively measured adherence.

With the advent of nebuliser devices (eTrack™, Pari and I-neb™, Phillips Respironics) that record time and date stamped treatments and support data transfer, we now have a means to capture objective treatment adherence data. This is important not only as an outcome measure for any intervention, but also to inform patients and clinicians of current adherence, given evidence of the effectiveness of feedback in order to change adherence behaviour [19, 20]. A key aim of the research programme was to develop a digital platform that could capture and display objectively measured nebuliser adherence and ‘make adherence visible’ and then to develop an associated behaviour change intervention to promote and support increases in adherence and the maintenance of adherence in the longer term. This paper describes the process of the development of the CFHealthHub digital platform and the associated behaviour change intervention to support adherence to nebulised treatment in adults with CF.

## Intervention development approach

The approach to intervention development that we employed was the combined approach identified in a taxonomy of intervention development; a ‘theory and evidence-based approach’ with a ‘target population-based’ approach [21]. The Behaviour Change Wheel (BCW) [22, 23] is a theory and evidence-based approach [20] selected because of the need to change the behaviour of people with CF. The person-based approach (PBA) to intervention development [24] is a target population-based approach [21] in which feedback from the target population is collected. It is complementary to BCW [24] and has been previously used alongside the BCW [25].

The BCW was devised following a systematic evaluation and synthesis of 19 frameworks of behaviour change and considers Capability, Opportunity, and Motivation in relation to Behaviour (COM-B model) [22]. The approach follows three stages: (i) Understanding the behaviour, identifying clear and specific target behaviours, and analysing the factors that impact on that behaviour and the need for change. This stage often uses the complementary Theoretical Domains Framework (TDF) [26, 27] that specifies 14 key domains from 33 behaviour change theories, that each influence capability, opportunity, or motivation [22, 28]. (ii) Identifying intervention functions and policy categories, i.e. ways to enact interventions, to achieve behaviour change, and (iii) identifying specific behaviour change techniques (BCTs), i.e. the specific active ingredients to change behaviour as described in the behaviour change technique taxonomy [29], and modes of delivery. The approach incorporates a systematic assessment of the available options and choices and has been widely used in the development of behaviour change interventions in settings including adherence (e.g. [30, 31]).

The PBA was devised from experience of developing digital interventions and utilises mixed methods with people from the target population to inform all stages of the intervention development in an iterative process. Given that we started with a plan to include a digital platform to display adherence data, PBA was an appropriate approach to the development of this digital intervention.

### Conceptual framework and aims of the intervention

We started this intervention development process with some initial ideas about what the intervention might include and the kinds of resources we might have to deliver it. Early work by the team using quality improvement and process mapping [32] had highlighted the need for objective data on medication adherence in CF, and we explored how this could positively impact on clinical practice [33–35]. Therefore, we aimed to develop a digital platform that could capture and display objective nebuliser adherence data to patients and clinicians. We understood the important role that the clinical teams play in CF and that adherence support and therefore intervention delivery would be supported by a trained healthcare professional [34].

**Fig. 1:**
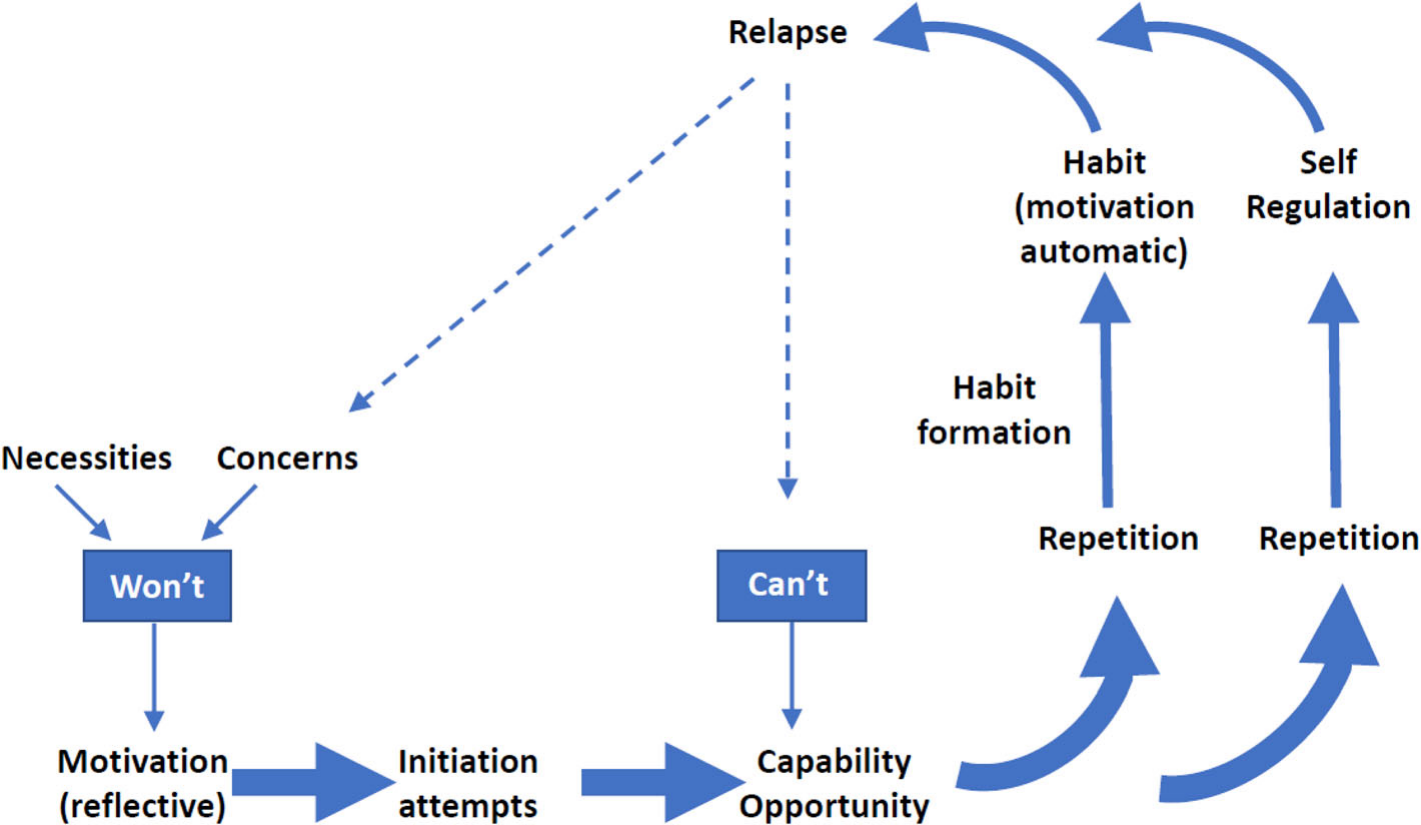
Conceptual framework for sustained adherence to treatment

Early work by members of the team [36, 37] had also considered barriers to adherence in CF and this fed into a conceptual framework of the broad factors influencing adherence and how an intervention might act on these factors to produce and then maintain change. A particular focus of this early work was a consideration of how adherence could be maintained without increasing perceived effort or burden. This conceptual framework drew on the COM-B model and also on other models of adherence and behaviour change and is presented in Fig. 1 (and see [22] p. 81).

The conceptual framework proposes that adherence behaviour is influenced by reflective motivation, i.e. a rational weighing up of the perceived necessity against the perceived concerns about treatment [38]. For some people, an intervention would need to address motivation before any other strategies could be successful since without this people with CF would not start to initiate attempts to adhere to treatment. Those who want to increase adherence to treatment will make attempts to do so but in many cases these attempts will be hindered by a range of capability and opportunity barriers. An intervention needs to support people to overcome these barriers so that they can adhere to their treatment. Self-regulation is one way in which people can sustain the life-long adherence to preventative inhaled treatment required to maintain lung health. However, there is evidence that self-regulation is difficult to maintain [39] and requires effortful self-control [40] and self-regulatory capacity [41]. Habit theory [42] proposes that habits formed through regular repetition of a specific behaviour in response to a cue over time (initially maintained through self-regulation) comes to trigger the behaviour (automatic motivation) such that habit strength then predicts the likelihood of the behaviour and motivation-driven self-regulation becomes less important. Habits have been proposed to be one of the key mechanisms by which behaviour change can be maintained in the longer term [43] with less perceived effort and burden, and thus a key aim of the intervention is to promote habit formation.

Having a conceptual framework from the start of the project provided a structure that guided intervention development. We recognised the potentially important role of capability, opportunity, and motivation and the overall aim of the intervention. However, we needed to understand the specific barriers and facilitators to adherence for people with CF and how we could best develop an intervention within this conceptual framework that would enable people to adhere and make habits.

## Methods

### The team

The core intervention development team included people with different perspectives, skills, and expertise: MA is a health psychologist with expertise in the development of theory-based behaviour change interventions. PW is a computer scientist and health informatician with expertise in the design and development of digital health platforms. SD and AOC are health services researchers with experience in undertaking qualitative research with behaviour change interventions. MH and JB are research physiotherapists with expertise in respiratory health and supporting patients with CF with their adherence. MW is a consultant in respiratory medicine working with adults with CF and with expertise in quality improvement. DB has cystic fibrosis and is a health services researcher and co-ordinated the patient and public involvement (PPI) group.

### Dynamic and iterative approach

**Fig. 2:**
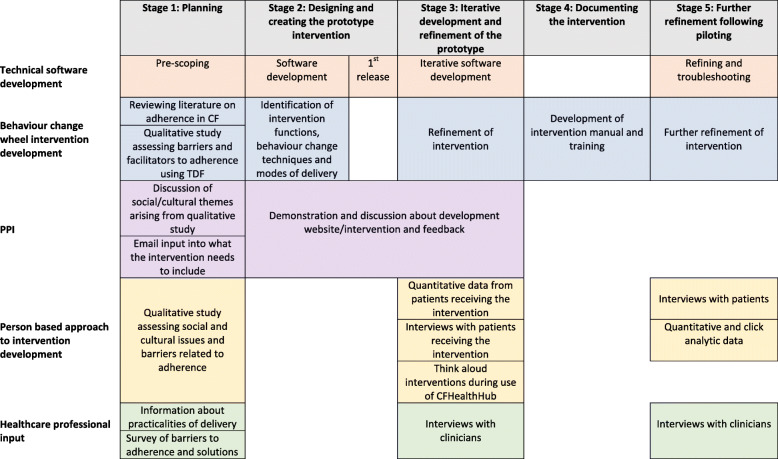
Process of intervention development

Intervention development is not a simple linear process. Different methods and actions are taken at different stages but they are used in a dynamic way in that they overlap and are revisited throughout the process [44]. The team followed an intervention development process with stages that fed into each other as illustrated in Fig. 2. Software development used the Agile process [45]. This involved the continuous delivery of working software to meet the shifting requirements identified by the intervention development team. The process required close collaboration between the technical and intervention development teams.

Ethical approval was gained for all studies [REC references: 15/YH/0332; 15/WS/0089] and all participants gave written informed consent.

## Stages of development

There are seven identified domains of actions taken across different approaches to intervention development: conception, planning, designing, creating, refining, documenting, and planning for future evaluation [21].

### Stage 1: Planning

#### Understanding practicalities of delivery

Input from members of the team working in the NHS context enabled us to understand that the intervention would need to be delivered flexibly by different members of the multidisciplinary team (MDT) or by health professionals recruited from outside of the MDT, due to NHS shortages in staff [46]. Thus, the intervention that was developed needed to be able to be delivered by a range of health professionals in order to ensure that future implementation was feasible.

#### Understanding the behaviour

We undertook a needs analysis for the intervention informed by the following.

##### Literature review

We reviewed the literature to identify key barriers to nebuliser adherence in adults with CF. This included a systematic review of qualitative studies [47], and we updated our knowledge with key papers published during the course of the development phase (e.g. [48]).

##### Qualitative research with patients

We conducted semi-structured interviews with 18 adults with cystic fibrosis from a single CF centre in the UK sampled by objective adherence, gender, age, and deprivation index. The data-prompted interviews [49] included the presentation of a graph showing each person their nebuliser adherence data over the last 6 months. The topic guide was informed by the literature review and based around understanding adherence in the context of cystic fibrosis and life in general, and the COM-B model [22] and TDF [26]. The data from these interviews were analysed using two different approaches; a framework analysis using the TDF, including a comparison of factors identified by higher and lower adherence [50], and a discursive analysis [51].

##### Survey with health professionals

We consulted health professionals to understand their perceptions of the barriers to adherence and possible solutions to address those barriers. Fourteen clinicians working at five adult CF centres across the UK were sent an email survey which was completed and returned by six clinicians.

##### PPI

Findings and interpretation of the interviews were provided to the patient co-applicant who led the PPI group to ensure that they were plausible and realistic. DB was involved from a very early stage in discussions around the proposed intervention, including the rationale for its use, as well as the design and functionality of the proposed website.

### Stage 2: Designing and creating the prototype intervention

Stage two of the intervention development process involved the development of a prototype intervention. There were two parts to the CFHealthHub intervention: (i) the digital platform displaying adherence data and online content and tools and (ii) the interventionist-delivered aspects of the intervention delivered during contact with a health professional. Frequent meetings of the intervention development team, informed by parallel discussions of the patient and public involvement group, were held during stages 2 and 3. At these meetings, we discussed input from each of the following activities.

#### Design of the prototype intervention using the Behaviour Change Wheel approach

Following the Behaviour Change Wheel approach^1^, we mapped intervention functions and behaviour change techniques to the identified needs of the intervention. Options were considered and discussed during meetings of the intervention development team, and decisions were informed by the APEASE criteria [23]: affordability refers to the cost of the intervention, which must be within budget; practicability refers to the extent to which the intervention can be delivered as designed to the target population; effectiveness/cost-effectiveness refers to effectiveness of the intervention in a real-world context in relation to that which is most cost-effective; acceptability refers to the extent to which the intervention is judged to be appropriate by different stakeholders; side-effects/safety includes unintended consequences of the intervention; and equity refers to the extent to which the intervention impacts on disparities in living standards, health, and wellbeing. We also considered the mode of delivery of each of the behaviour change techniques, whether they were delivered via the digital platform or whether they were delivered by a health professional acting as an interventionist. MH, a physiotherapist experienced in the delivery of adherence support in CF care, informed the development of the interventionist-delivered components.

#### Design of the CFHealthHub digital platform

**Fig. 3:**
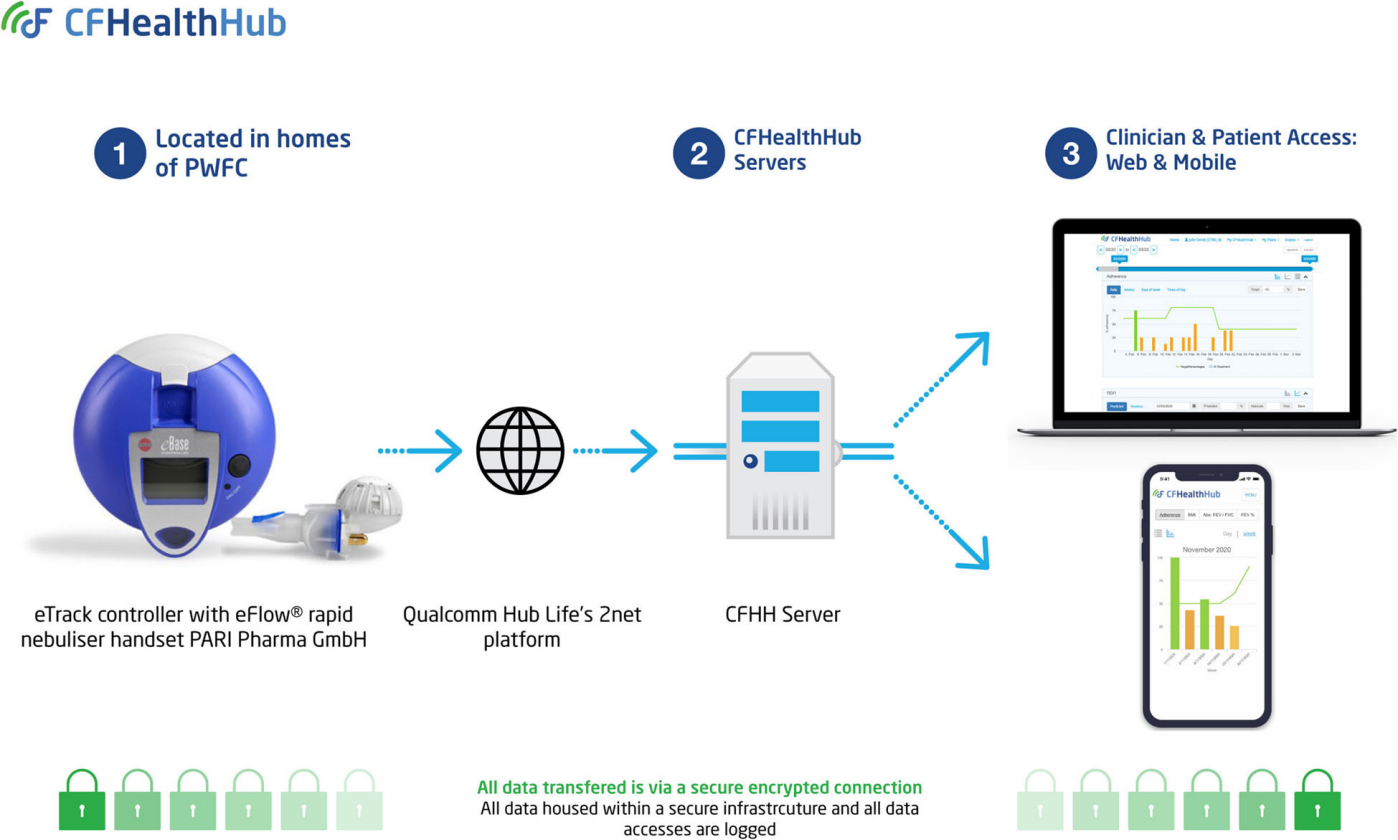
Data transfer flow

The first phase of technical development was to develop the process and mechanisms by which inhalation data (time-stamped nebulisations) could be automatically captured from third party devices and software, transferred securely, and displayed in a usable way on a digital platform in relation to prescription data (i.e. treatment taken/treatment prescribed). The data transfer flow is shown in Fig. 3. From the eTrack nebuliser, inhalation data was automatically sent to a Qualcomm Life 2net Hub located in the participant’s home. Data was transferred from the Qualcomm Hub to a secure server maintained by Pari and then forwarded on to the CFHealthHub server for display and use in the CFHealthHub digital platform. Data transfer was in real-time and required no additional actions by the participant over and above normal nebuliser usage, assuming all devices maintained connectivity with the required networks. This phase also involved a 6-week development and testing phase, where the data transfer mechanism was tested, and the data quality of the transmitted data was validated (see Fig. 2, prototype intervention 1). This was refined over a number of iterations. The need for specific content and tools that arose from the intervention development work fed into further technical developments and prototype releases.

### Stage 3: Iterative refinement of the prototype using the person-based approach

Five participants were people with CF who were aged 16 or older, on the CF registry, provided with an eTrack nebuliser and Qualcomm hub, and given access to the CFHealthHub platform in order to assess the ability of the system to successfully record and display nebulisations. They were followed up after 1 week to troubleshoot any data upload issues and interviewed after a period of 1 month to give them time to use the prototype intervention.

Twenty-two participants were recruited into the iterative development study. Participants were people with CF who were aged 16 or older, on the CF registry, and willing to take inhaled mucolytics via a chipped nebuliser (eTrack). They were provided with an eTrack nebuliser and Qualcomm hub. They received four sessions of intervention delivery from a physiotherapist and were given independent access to the CFHealthHub web platform. We conducted 18 semi-structured telephone interviews with participants in different cycles of the software development (see Fig. 2, protoypes 1–5) to ask about acceptability of the appearance and functionality of the digital platform and potential improvements. Additionally, we conducted six in-depth think aloud interviews [52, 53] with participants whilst they were using a version of the CFHealthHub website. The screen and audio of the interview was recorded using Camtasia™ software. This allowed the software team to identify technical and navigational issues with the website that were corrected in subsequent releases. We also interviewed the physiotherapist delivering the intervention at two time points for wider views about how to deliver the intervention to patients and how that linked to the clinician view of the website.

#### PPI

Input was provided by DB and wider PPI reference group throughout the early development phase of the intervention. Initially, this involved providing input into the proposed content for the textual parts of the website, in terms of the type and level of information that was felt appropriate, not only for people with CF, but others involved in their care.

As the digital platform was developed, PPI input was again provided at regular intervals. Members of the PPI reference group were given the opportunity, on a number of separate occasions, to explore iterations of the website through a demonstration version of the website. Feedback was then provided in meetings of the group, which was then passed back to the wider study team.

Aside from input on the design, group members also provided comment on practical issues around data sharing within the website, and the user guide that had been produced to accompany it.

### Stage 4: Documenting the intervention

At the end of this process, in readiness for the pilot trial, we created an intervention manual that outlined the key components of the intervention, how to use the CFHealthHub digital platform, and the manner and structure of delivery, and an associated training programme for interventionists as well as a user guide for participants.

### Stage 5: Further refinement of the intervention following piloting

Whilst descriptions of the intervention development process often stop before piloting and feasibility testing [44, 54], we utilised the pilot and feasibility study [55] to identify further refinements that were made to the intervention before it was used in the final randomised controlled trial.

The pilot and feasibility study consisted of a mixed methods process evaluation undertaken concurrently with a pilot RCT in two UK CF centres. Participants were people with CF who were aged 16 or older, on the CF registry, and willing to take inhaled mucolytics and/or antibiotics via an eTrack nebuliser. Three interventionists were trained to deliver the intervention in six face-to-face meetings over 5 months to 32 participants who had access to the CFHealthHub (CFHH) website throughout. We conducted 25 semi-structured face-to-face interviews with patients in the intervention arm of the RCT (*n* = 14), interventionists delivering the intervention (*n* = 3 at 2 time points), and members of the wider multidisciplinary team (MDT) (*n* = 5).

The findings from the quantitative [55] and qualitative [56] aspects of the study were triangulated [57] and the implications for the further refinement of the intervention discussed by the development team with input from PPI representatives. As the software was more mature at this stage, changes became more costly in terms of implementation effort and therefore regular prioritisation meetings were conducted where the team agreed on which requirements would be implemented. Decisions about which work to prioritise were made using the MoSCoW criteria of prioritisation: Must have, Should have, Could have, and Won’t have [58].

A full description of the intervention was written following the TiDieR (Template for Intervention Description and Replication) checklist [59].

## Results

### Stage 1: Planning the intervention

The research confirmed that different factors influenced different people’s ability to adhere and it was therefore important, for reasons of equity, to develop an intervention that addressed multiple capability, opportunity, and motivation barriers to adherence. Our behavioural needs analysis enabled us to identify the factors that the intervention needed to address (see Table 1) and the team considered and discussed whether the intervention should be designed to address each of these needs. Three domains were excluded at this stage (see Table 1 for rationale).

**Table 1:** Needs analysis by COM-B and TDF with decisions for inclusion/exclusion in the intervention

COM-B component	TDF domain	Needs analysis	Inclusion/exclusion
Physical capability	Physical skills	Need skills to use nebuliser, prepare treatment, and clean nebuliser	Included
Psychological capability	Knowledge	Need to know about the correct treatment-taking procedures, to understand treatment action and the importance of nebuliser treatment Need to address treatment concerns	Included
	Memory, attention, and decision processes	Need to remember to take treatment	Included
	Behavioural regulation	Need to develop routines, plans, and habits for treatment Need to monitor adherence behaviour	Included
Physical opportunity	Environmental context and resources	Need to have a time, place, and the equipment do take treatment Need to develop strategies to take treatment around specific barriers or times when treatment taking is more difficult	Included
Social opportunity	Social influences	Need positive support from family, healthcare professionals, and/or others to do treatment Need to have social norms for adherence	Included
Reflective motivation	Professional/social role and identity	Need to develop a social identify that is consistent with treatment adherence	Excluded—social identity change in the context of adherence is not well understood
	Beliefs about capabilities	Need to develop confidence in the ability to take and adhere to treatment Need to develop strategies to take treatment at times or in situations when it is more difficult	Included
	Optimism	Need to be optimistic that full treatment adherence is possible	Excluded—focus on achieving higher but realistic adherence
	Beliefs about consequences	Need to believe that treatment is effective and necessary for long-term health, symptom reduction, avoiding IVs/hospital stays Need to believe that treatment is important irrelevant of perceptions of current wellness Need to address treatment concerns	Included
	Intentions	Need to encourage intentions to adhere to treatment and avoid periods of intentional non-adherence	Included
	Goals	Need goals for treatment adherence Need to develop strategies to address conflicting goals	Included
Automatic motivation	Reinforcement	Need to provide some reinforcement for treatment-taking Need to address treatment concerns	Included
	Emotion	Need to focus on emotional drivers for adherence (fear, anticipated regret) Need to address low mood and avoidance	Excluded—not acceptable to focus on fear and may increase avoidance. Addressing low mood/depression outside the scope of this intervention (signposting to other support services)

### Stage 2: Designing and creating the intervention

The stage 1 analysis indicated that a number of needs were replicated across different TDF domains (e.g. need to address treatment concerns), and the intervention development team therefore generated ‘themes of need’ for the intervention (see Table 2). The selection of intervention functions matched to each theme of needs is described in Table 2 along with the reasons for inclusion/exclusion according to the APEASE criteria. Table 2 also displays the selection of behaviour change techniques (BCTs) to match the needs and intervention functions selected. BCTs that were considered but rejected by the team according to the APEASE criteria are also shown.

**Table 2:** Intervention themes, potential, and selected intervention functions, and potential and selected behaviour change techniques (BCTs)

Intervention themes (and associated TDF domains)	Needs addressed within module	Intervention functions included with description of the context of use	Intervention functions considered and rejected with APEASE rationale	BCTs selected; for full descriptions of BCTs see [29]	BCTs considered and rejected with APEASE rationale
A need to understand treatment (physical skills, knowledge, beliefs about consequences, intentions)	Need to know about the correct treatment-taking procedures, to understand treatment action and the importance of nebuliser treatment Need to address treatment concerns Need to believe that treatment is effective and necessary for long-term health, symptom reduction, avoiding IVs/hospital stays Need to believe that treatment is important irrelevant of perceptions of current wellness Need to encourage intentions to adhere to treatment and avoid periods of intentional non-adherence	Education (knowledge on the importance of treatment-taking even when well, effectiveness of treatment, and treatment action) Persuasion (using imagery and other communications strategies to persuade about the importance of consistent long-term adherence) Modelling (peers who have knowledge and understanding about nebuliser treatment and how they adhere)	Coercion—not acceptable to patients or health professionals to focus on punishment for non-adherence	5.1 Information about health consequences 9.1 Credible source 5.2 Salience of consequences 6.1 Demonstration of the behaviour 16.3 Vicarious consequences 15.4 Self-talk	5.5 Anticipated regret: potentially inducing fear not deemed acceptable (acceptability) 9.2 Pros and cons: Not practical as would take too long to deliver within the intervention (practical) 9.3 Comparative imagining of future outcomes: may be challenging for some patients and could induce fear given life-limiting nature of CF (practical, side-effects, equity).
A need to be able to monitor adherence (behavioural regulation)	Need to monitor adherence behaviour and outcomes	Education (knowledge about own adherence data) Environmental restructuring (providing a nebuliser, a hub, and a digital platform to track and provide data on nebuliser adherence) Enablement (providing behavioural support to provide feedback on adherence data)		2.3 Self-monitoring of behaviour 12.5 Adding objects to the environment	2.4 Self-monitoring of outcomes of behaviour: no easy mechanism to monitor symptoms or health and changes may be due to factors other than adherence (practical, side-effects)
A need to have treatment goals and feedback (goals, reinforcement)	Need goals for treatment adherence Need to provide some reinforcement for treatment-taking	Enablement (providing behavioural support to set realistic specific goals for treatment) Incentivisation (creating an expectation of rewards when goals are met)	Coercion—not acceptable to patients or health professionals to focus on punishment for not meeting goals Modelling—not clear what feedback on others behaviour might be most effective to produce change, and could backfire	1.1 Goal setting (behaviour) 2.2 Feedback on behaviour 1.6 Discrepancy between current behaviour and goal 1.5 Review behaviour goal 8.7 Graded tasks 10.4 Social reward	1.3 Goal setting (outcome): the achievement of some outcome goals could be impacted on by factors outside of the individual’s control and could result in demotivation (side-effects)
A need to have confidence in the ability to adhere to treatment (beliefs about capability, social opportunity)	Need to have social norms for adherence Need to develop confidence in the ability to take and adhere to treatment	Modelling (providing role models of people who have increased their adherence for people to aspire to) Persuasion (using communication to increase feelings of positive self-efficacy)	Environmental restructuring—not practical to change the environmental barriers that make treatments feel difficult to do Education—not likely to be effective given that confidence likely to be based on past experiences of trying to adhere and potentially failing.	6.1 Demonstration of behaviour 15.1 Verbal persuasion about capability 15.3 Focus on past success	6.2 Social comparison: drawing attention to others (natural) adherence behaviour could create social norms for non-adherence given low median levels (side-effects)
A need to have a treatment plan (behavioural regulation, memory, attention, and decision processes)	Need to remember to take treatment Need to develop routines, plans and habits for treatment	Environmental restructuring (providing digital tools on which to make and record plans) Enablement (providing behavioural support to identify appropriate plans)		1.4 Action planning 8.3 Habit formation 7.1 Prompts/cues	1.8 Behavioural contract: writing and signing contract would take too much time (practical) 15.2 Mental rehearsal of successful performance: not likely to be effective given that it is not treatment taking that is the challenge but adherence in a range of contexts (effective)
A need to solve problems around treatment adherence (environmental context and resources, goals, social opportunities, beliefs about capability)	Need skills to use nebuliser, prepare treatment, and clean nebuliser Need to have a time, place and the equipment do take treatment Need to develop strategies to take treatment around specific barriers or times when treatment-taking is more difficult Need to develop strategies to address conflicting goals Need positive support from family, healthcare professionals and/or others to do treatment Need to develop strategies to take treatment at times or in situations when it is more difficult	Training (skills to be able to adhere use and clean nebuliser and mix treatment) Education (providing knowledge about support services and strategies to overcome barriers to treatment-taking) Environmental restructuring (providing digital tools on which to make and record plans, and provide educational information, directly changing the environmental context and resources for some patients where possible) Enablement (providing behavioural support to identify barriers and make plans about how to overcome them or reduce their effects) Modelling (role models of others who have developed strategies to overcome barriers to adherence)	Restriction—not acceptable to require patients to focus on treatment and reduce opportunity to engage in behaviours associated with other goals	1.2 Problem solving 12.1 Restructure the physical environment 15.4 self-talk 3.2 social support (practical) 4.1 Instruction on how to perform the behaviour 6.1 Demonstration of the behaviour 8.1 Behavioural practice/rehearsal	9.3 Comparative imaginings of future outcomes (practical)

Discussions about the intervention considered the needs of different types of patients with different barriers to adherence, as indicated in the analysis of the stage 1 qualitative work. We therefore considered how the intervention could be tailored to meet the needs of individuals and to reduce the possibility of overwhelming an individual with lots of BCTs that might not be useful or relevant. There were two key aspects of tailoring that we incorporated early on: modules of content and paths through the intervention. In relation to the *need to understand treatment*, people had a range of necessity and concern beliefs about the treatments that they had been prescribed, and we only needed to address their specific beliefs related to lower adherence. To address this, we identified the need for a personalised area where we could locate specific targeted and tailored content which we named the *Toolkit*. Participants could access their toolkit directly from the home page. Educational and persuasive content was grouped into six themed modules of content informed by the literature and our qualitative work, e.g. *Why is it important that I do my nebuliser treatments every day?* and *I have concerns about my nebuliser treatments*. We devised an algorithm to automatically prioritise up to three modules of content for each participant based on their responses to matched items in the BMQ-Specific^2^ [38]. For example, a ‘strongly disagree’ response to the beliefs statement, *This nebuliser treatment protects me from becoming worse,* made it more likely that they would be matched to the *I’m not convinced that my nebuliser treatment works* module. Interventionists could override and change modules if discussions with participants indicated other or changed priorities over the course of intervention delivery identified during review sessions. Following consultations, interventionists could also select specific modules of problem-solving content and videos matched to the specific needs of the participant and place these in the Toolkit area.

The educational/persuasive content of the modules was created to ensure that the information was accessible and meaningful to people with different needs and different perceptions of ‘credible sources’. It included simple animated videos of treatment action, patient stories and links to external websites (e.g. CF Trust, NHS) and Cochrane reviews about drug treatments. All of the content was reviewed by the PPI groups and feedback was sought throughout the iterative development process.

We understood that setting specific goals and plans for treatment would only be effective if people were sufficiently motivated to increase their adherence, and if not, then the intervention should follow a different path that replaced goal setting/planning with ensuring a *need to understand treatment* and *confidence building* using the modules and videos available on CFHealthHub and open, non-judgmental discussions with patients.

Some aspects of the intervention were less about the tools and content of the CFHH website and more about how the interventionists interacted with participants. For example, the self-efficacy intervention components required that interventionists focused on times when treatment had been taken rather than times of non-adherence during discussions about treatment graphs.

### Stage 3: Refining the intervention

Feedback from discussions about the behaviour change techniques and the iterative development study fed into both the technical development of the CFHealthHub digital platform and how the intervention would be delivered by health professionals (the interventionist-delivered components). The versions of CFHealthHub that the participants received changed over time as new content and tools were added into the digital platform (see Table 3). Table 4 provides the main feedback from participants and interventionists from this process along with how these were responded to in the development process.

**Table 3:** Key components of prototype CFHealthHub digital platform versions 1–5

Version	Key components
1	Basic adherence display (blue) + basic prescription entry
2	First behaviour change intervention release: includes *my treatment*, *problem solving*, and *my toolkit* Allows clinicians to add/edit patients Basic patient analytics included (page clicks)
3	*Action plan* tool Adherence display updates to show treatment times in labels Enhanced analytics to capture graph views and clicks
4	Updated designs for *my treatment* (more lively colours and images) 24-hour clock used in labels on graphs for clarity about timing of treatments
5	Clinician report page added Screening tool added to tailor *my treatment* *Coping plans* added Finalised revised design and content of *my treatment* Revised design and content of *problem solving* Revised design and content of *my toolkit*

**Table 4:** Key feedback and decisions from qualitative interviews conducted during iterative development cycles

Feedback	Make changes	Rationale/comments
Participants thought blue adherence graphs look and homepage looked too ‘NHS’ and boring.	Accept	Changed design to look more distinctly CFHealthHub. More images
Some participants liked the picture on the homepage, other did not. There was no consensus about which image was preferable. Some participants requested to personalise with their own images.	Accept	Needs to feel like it belongs to the participant. There is no one image that meets the needs of everyone. Images could be of a goal (e.g. upcoming holiday) which could serve to act as a motivator
Participants wanted a forum or some way to interact with other people with CF, to share experiences, problems, and ideas.	Reject	Concern that this would require moderation (not practical) and that, given that low adherence is very common, could serve to normalise non-adherence and demotivate participants. Instead decided to incorporate ‘talking heads’ video clips—providing some information from others with CF but where we could control the content.
Participants want to instantly see achievements/progress on graphs. Suggested traffic light system. Wanted to see data over a longer time frame.	Accept	Some concern about traffic light system—specifically that too much red would put people off. But suggestion came up frequently. Modified over iterations so that green = hit target, amber = some treatment but not met target, red date = not treatment taken. Participants able to open out data to look over a longer period.
Participants confused by prescription entry. Suggested making this clinician entered with mechanism by which participants can flag if their prescription changes.	Accept	Correct prescription data is vital to success of the intervention as this is what the adherence data is based on. Important to get right.
Clinicians suggested that it would be useful to be able to see treatment times easily, e.g. when you hover over a bar on the charts.	Accept	Clear benefit for both patients and clinicians.
Some participants go to bed late and their last treatment appears on the following day’s graph. Would be useful to be able to modify the time for the end of day to adjust this to meet different lifestyles of patients	Reject	Extensive programming task to change the basic time set up from midnight-midnight day.

### Stage 4: Documenting the intervention

The final intervention was documented in a manual for interventionists. This included the following sections that focused on motivating health professionals about the value of the intervention as well as the knowledge and skills that they needed to deliver it:

- Description of the overall intervention and the rationale for its development
- Orientation to the CFHealthHub digital platform including the data displays, behaviour change content and tools, and how to use them
- Plans for different types of intervention delivery sessions including how to prepare for them
- Information about how to tailor the intervention to suit different patients’ needs
- Intervention delivery using a person-centred approach

An associated training programme for interventionists was developed.

### Stage 5: Further refinement following piloting

The outcomes of the pilot and feasibility study are described in detail elsewhere [55, 56]. Key outcomes that fed into further development of the intervention and how they were responded to in subsequent developments are described in Table 5, and the final intervention that was used in the randomised controlled trial are described in Table 6.

**Table 5:** Key feedback and decisions from qualitative interviews conducted during pilot and feasibility study

Feedback	Make changes	Rationale/comments
Limited engagement with CFHealthHub outside of visits with the interventionist	Accept	To address this, we prioritised the development of the CFF app for the trial (must have) and we added tools to encourage engagement, i.e. push notifications sent from the app each Monday congratulating participants on meeting their target or encouraging them to start again. We also added a reminder message sent if CFHH had not been accessed for a period of 2 weeks.
Fewer interventionist sessions were delivered than anticipated	Accept	We devised a clear intervention pathway that indicated the frequency, interval, and pattern of intervention sessions for each participant for the trial.
Limited delivery of some key BCTs by interventionists	Accept	Modify interventionist training and handbook and create worksheets to follow. Monitor delivery of key BCTs during trial.
Changes to the target line changed the target line for all time periods	Accept	Keep and show historical target lines, and traffic light system in response to the specific targets at a specific time-point.
Lack of faith in the validity of the adherence data by participants and interventionists	Accept	Some initial problems with the pairing of devices caused some issues with data at the beginning of the pilot trial. High quality control to ensure effective pairing. Some cases the data appeared correct. Created further training and protocols on missing data and how interventionists should response to scenarios in which the validity of data was questioned.
Some participants did not want to watch videos of other people with CF. Making social comparisons was threatening.	Accept	Inclusion of ‘talking heads’ videos made an optional part of the intervention.

**Table 6:** Final intervention BCTs and mode of delivery

Module	BCTs	Mode of delivery
My treatment	4.1 Instruction on how to perform the behaviour 5.1 Information about health consequences 9.1 Credible source 5.2 Salience of consequences 6.1 Demonstration of the behaviour 16.3 Vicarious consequences 15.4 Self-talk	*CFHealthHub*: • Demonstration of techniques for nebuliser use, cleaning and treatment mixing • Information about CF, the need for treatment, how each treatment works, and the importance of adherence • Information presented in a variety of ways though written text, patient stories, ‘talking heads’, and animation videos, with links to external content including Cochrane reviews • Range of different credible information sources including PWCF, Clinicians, links to scientific papers *Interventionist*: • Interventionist introducing and highlighting relevant content on CFHealthHub • Interventionist eliciting self-talk through discussion of motivation
Self-monitoring	2.2 Feedback on behaviour 2.3 Self-monitoring of behaviour 12.5 Adding objects to the environment	*CFHealthHub* • Charts and tables of objective adherence data presented within CFHealthHub *Interventionist* • Introducing and explaining charts and tables to participants
Confidence building	6.1 Demonstration of behaviour 15.1 Verbal persuasion about capability 15.3 Focus on past success	*CFHealthHub* • ‘Talking heads’ videos of coping stories within CFHealthHub *Interventionist* • Interventionist encouraging focus on periods of higher adherence on charts
Goal setting and review	1.1 Goal setting (behaviour) 2.2 Feedback on behaviour 1.6 Discrepancy between current behaviour and goal 1.5 Review behaviour goal 8.7 Graded tasks 10.4 Social reward	*CFHealthHub* • Indication of goal line on charts of adherence • Visual indication of goal met on CFHealthHub • (Optional) Weekly push notifications indicating whether goal was met • (Optional) Reward messages sent when goal met *Interventionist* • Discussion and agreement of goals with interventionist • Review of goals • Suggested steady increase in goal as improvements are made • Feedback and social reward on progress
Treatment Plan	1.4 Action planning 8.3 Habit formation 7.1 Prompts/cues	*CFHealthHub* • Action planning tool and storage within CFHealthHub *Interventionist* • Help to focus on identifying consistent cues and linking to behaviour (habit formation) • Discussion and identification of appropriate cues - and how to add to the environment (if necessary)
Problem-solving	1.2 Problem solving 12.1 Restructure the physical environment 15.4 Self-talk 3.2 Social support (practical) 4.1 Instruction on how to perform the behaviour 6.1 Demonstration of the behaviour 8.1 Behavioural practice/rehearsal	*CFHealthHub* • Solution bank within CFHealthhub (including advice to problem solve, restructure the physical environment, engage social support) • Coping planning, day planner, and party planner tools and storage within CFHealthHub • Videos demonstrating correct use of nebulisers within CFHealthHub *Interventionist* • Tailored problem-solving guided by interventionist • Support to create day plans/party plans where appropriate • Support to construct if-then coping plans including identifying self-talk where appropriate

The intervention manual was revised to address the identified need for change and to include information about the CFHealthHub mobile apps (iOS and Android) and associated functions. We developed worksheets for interventionists to follow during delivery of the intervention sessions. These worksheets included step by step instructions about how to interact with the CFHH content and the participant and included hints about how to phrase questions. An example worksheet for the first intervention visit is included in supplementary files. The associated training programme for interventionists was revised to be delivered in 2 face-to-face training days with 4 days of independent online training delivered via a virtual learning environment and ongoing tutorial support.

Modifications and adjustments to how the intervention was tailored and personalised were also considered and these are described in Tables 7 and 8.

**Table 7:** Tailoring of the intervention components to meet specific participant needs

Tailored component	How are non-tailored components accessed	How version is determined
Contents of ‘My treatment’ and ‘Problem-solving’ focus on information relevant to current prescription drugs.	All generic information in available to all participants to browse. Information on treatments not currently prescribed are available but minimised.	Prescription is entered into CFHealthHub at consent and altered whenever there is a prescription change. CFHealthhub automatically tailors content based on this information.
Modules of ‘My treatment’ are selected and placed into ‘My Toolkit’ based on the scores on the BMQ-Specific [38] questionnaire with one additional item^a.^	Participants can browse all modules of ‘My treatment’.	Participants’ responses to the BMQ questionnaire [38] are entered into CFHealthHub at consent. CFHealthHub recommends the most relevant modules based on a scoring algorithm. If CFHealthHub recommends more than three modules then interventionists select three based on the scores and their judgement based on conversations with the participant. Modules can be changed throughout the intervention and these are recorded via CFHealthHub.
Modules of ‘Problem-solving’ are selected and placed into ‘My Toolkit’ based on the barriers identified in consultations with the interventionist.	Participants can browse all modules of ‘Problem-solving’.	Interventionists can select modules of problem-solving content based on the barriers identified in consultations. Modules can be changed throughout the intervention and these are recorded via CFHealthHub.
‘Talking heads’ videos are selected to match key participant characteristics and placed into ‘My Toolkit’. This is optional.	Participants can browse the entire ‘talking heads’ video library.	Interventionists can select relevant videos that match key characteristics of the participant (e.g. age, gender, occupation, life role, problems experienced). Videos can be changed throughout the intervention and these are recorded via CFHealthHub.
Goal setting and review and treatment planning are only utilised for participants who are motivated (want to) take more treatment. Participants with very low motivation do not receive these parts of the intervention. Instead they spend more time focusing on the content of ‘My treatment’ and relationship building with the interventionist.	Participants can choose to set goals and make plans at any point in a consultation or by contacting the interventionist.	Very low motivation is determined by a combination of a low motivation score on a questionnaire item and discussion with the participant in a consultation. The identification of very low motivation is recorded where this applies.

^a^We added the following item to the BMQ-Specific questionnaire based on our qualitative work about treatment beliefs: *Nebulised antibiotic treatments are more important for my health than IV antibiotics* (strongly agree to strongly disagree)

**Table 8:** Personalisation of the Intervention

Personalised component	How personalisation is achieved
Graphs and charts show personal data	Participants eTrack nebuliser collects and send adherence data to CFHealthHub via the Qualcomm hub for display.
Target line on graph	Participants determine their adherence goal in consultation with the interventionist. This is displayed on their charts.
Plans	Participants make individual plans based on discussions with the interventionist. These are made using the tools within CFHealthHub and recorded in ‘My Toolkit’. New plans can be added and CFHealthHub records all plans for each participant.
Home page	Participants can select an image to display on their home page from a default selection, or can upload their own image.
Notifications	Participants can optionally choose to receive personalised notifications via the CFHealthHub app. These send a message to let the participant if they have met their goal in the previous week, they have met their goal on most days in the previous week, or an encouraging messaging to keep going if they did not.
Reminders	Participants can optionally choose to receive reminders via the CFHealthHub app. These send a reminder message if the participant has not accessed their CFHealthHub account for a period of 2 weeks.
Reward messages	Reward messages are displayed on the CFHealthHub website or mobile app following log-in if the participant had met their adherence goal for 4 out of the last 7 consecutive days. This reward is only shown if inhalation data has been received for the last 7 consecutive days.

## Discussion

The CFHealthHub intervention which comprised a digital platform and delivery by a health professional (with an associated manual and training) was developed through a rigorous and systematic development process. It was shown to be usable and acceptable to people with CF and the clinical community. Our development work demonstrated that inhalation data could be automatically transferred from a third party nebuliser device and displayed, in combination with prescription data, to provide visibility of a participant’s adherence in real-life settings. Participants and clinicians were able to understand and interpret the data display quickly and easily. The intervention includes behaviour change techniques to address adherence to nebuliser treatment in people with CF, which we have been able to tailor and personalise, so that it is appropriate for people with a range of different barriers to adherence.

A key value of our approach is that it has incorporated theory- and evidence-based approaches (Behaviour Change Wheel [22, 23]) and target population-based approaches (person-based approach [24, 31]) [21]. Throughout the development process, we have been responsive to feedback and have changed and refined the intervention and the way in which it is delivered. The iterative process allowed issues to be identified quickly and updated rapidly, often during the early design phases, which minimised the cost and resources required to make the changes. Whilst pilot and feasibility studies are often considered to be outside of the intervention development process [44, 54], we utilised this opportunity to make further refinements to the intervention and its implementation in readiness for the full randomised controlled trial. We anticipate that the refinement process will continue following the randomised controlled trial and process evaluation in which it is currently being tested (trial registration, ISRCTN55504164) to inform implementation in clinical practice (trial registration, ISRCTN14464661).

Models for intervention development offer a pathway for intervention development but not a solution. Multiple decisions have to be made along the way, and the way in which they are made and the rationale behind those decisions depends in part on the intervention development team and their skills, experiences and expertise. The APEASE criteria [22] informed decisions and MoSCoW [58] enabled us to prioritise later changes, but the information that fed into the assessment of those criteria required the expertise of the development team. Ideas that seemed practical from the perspective of a health psychologist were not always practical from the perspective of a computer scientist. In addition, in terms of the likely effectiveness of the selected components, this required reference to our conceptual framework (see Fig. 1) and a good understanding of the theories and principles that underpinned different aspects of it; thus, the intervention drew on a range of different theories and evidence. The *My treatment* module (see Table 7) reflected the necessity-concerns framework [38], given that knowledge and beliefs about the consequences of treatment largely related to perceptions about the necessities and concerns for nebuliser treatment and social cognitive theory [60, 61] in terms of the role of outcome expectancies in behaviour. *Confidence building* also drew on social cognitive theory, employing a number of established strategies to increase self-efficacy (mastery, vicarious experiences, etc.). The *self-monitoring, goal setting and planning* modules drew on control theory [62], to explain how increased awareness of adherence behaviour and the identification of a discrepancy between current behaviour and goal might result in self-regulated behaviour change through action planning. Habit theory [42, 63] influenced the structure of action planning used in the intervention, i.e. the identification of a cue or prompt for nebuliser treatment and the use of an implementation intention [64] *if cue then nebuliser treatment-*based action plan. Coping plans were used as part of *problem solving* [65] in order to address the issue of capability and opportunity barriers to treatment adherence and to help maintain adherence [43].

The development process was highly dependent on multidisciplinary working involving a team comprised of academics, clinicians, patients with CF, research software engineers, and UX designers. There were varying levels of understanding and experience of each other’s roles, working practices, and workload. The initial model was based on obtaining and analysing participant feedback, agreeing design and software development requirements followed by development sprints, giving a new release of CFHH for each cycle of participants. However, difficulties in providing qualitative feedback in a timely manner identified previously [66] and the time required for design changes, programming, and testing meant this was not a viable working model. Regular meetings to facilitate clear communication, less technical and clinical language, and patience were required in order to develop the intervention, and many of the desired changes required significant development time such that some of the changes identified in stage 2 of the process were not actually included in the CFHealthHub digital platform until the beginning of the RCT. We recommend that future projects have early and ongoing team discussions so that expectations from all involved parties are realistic.

### Limitations

There are some limitations of the approach. The data that informed stages 1 and 3 of the process stemmed from patients and clinicians from a single CF centre, and in stage 5 (the pilot study), from a further two centres and their associated clinical teams. This means that the intervention was based on a relatively small sample, and it is feasible that these centres may have differed from others in key ways, not least of which might have been high levels of motivation, and the clear commitment of the principal investigators at each site. The intervention development process was driven by a particular development team, made up of a particular mix of skills and expertise, and it is conceivable that a different team with different members may have made different decisions and arrived at a very different intervention. Whilst the input from patients, clinicians, and the multidisciplinary team was valuable, the process was resource and time intensive.

The intervention that we have developed is a complex one with multiple components tailored to meet the needs of different patients. Whilst this is a potential strength, it is also a potential limitation in that intervention delivery is quite long, and selection of the appropriate components for a particular patient relies on the skills and training of the interventionist. An alternative approach would have been to focus on one aspect of need (e.g. planning and habit formation) and develop an intervention just focused on these components. However, this would have meant that the intervention was not suitable for patients with lower motivation. Given that this group is of particular concern to clinicians (because they tend to be the least well), we felt that it would not be equitable or acceptable to design an intervention which did not target adherence increases across different patient groups. Following the RCT, we will be able to undertake analysis to explore which aspects of the intervention produced the intended changes in process outcomes and which did not, and if there are particular groups of patients for whom the intervention worked more or less well. This will enable us to pare down and refine the intervention further so that in the future, the intervention can be more tailored and can incorporate just those components found to be successful in improving adherence and this will likely reduce the length and complexity.

The initial qualitative work undertaken in stage 1 indicated that for patients, nebuliser use was seen as an integral part of their CF treatment alongside chest physiotherapy, diet, and enzymatic treatment for digestive issues, and for many patients, other treatments for co-morbid conditions including diabetes and liver conditions. It was beyond the scope of this programme of work to develop a system that could support adherence to all of these aspects of care.

## Conclusion

We have devised an intervention to increase adherence to nebuliser treatment in adults with CF with substantial input from patients and clinicians and which has a strong theoretical and evidence base. The intervention comprised a digital platform (www.cfhealthhub.com) and components delivered in patient consultations with an interventionist. It is usable and acceptable to people with CF and clinicians. The intervention is currently being tested in a randomised controlled trial across 19 UK sites.

## Supplementary Information

**Additional file 1.** First Intervention Visit: Worksheet

## Data Availability

The datasets used and/or analysed during the current study are available from the corresponding author on reasonable request.

## Abbreviations

APEASE: Affordability, Practicability, Effectiveness/Cost-effectiveness, Acceptability, Side-effects/Safety, Equity
BCTs: Behaviour change techniques
BCW: Behaviour Change Wheel
CF: Cystic fibrosis
CFHH: CFHealthHub
COM-B: Capability, opportunity, and motivation—behaviour
MDT: Multidisciplinary team
MoSCoW: Must have, should have, could have, won’t have
PBA: Person-based approach
PPI: Patient and public involvement
RCT: Randomised controlled trial
TiDieR: Template for Intervention Description and Replication
TDF: Theoretical domains framework

## Footnotes

1 We omitted the selection of policy categories stage of the BCW process as the decision to focus on service provision had been made previously during the development of the programme aims.

2 We also included an additional belief item identified as important in our qualitative work: *Nebulised antibiotic treatments are more important for my health than IV antibiotics*
