# Supplementary material for: Development of an intervention to increase adherence to nebuliser treatment in adults with cystic fibrosis: CFHealthHub
Source: Pilot Feasibility Stud. 2021 Jan 4;7:1. doi: 10.1186/s40814-020-00739-2 (PMC7780635; doi:10.1186/s40814-020-00739-2)
Supplement: Supplementary file 1 — Additional file 1. First Intervention Visit: Worksheet [file 40814_2020_739_MOESM1_ESM.pdf]

First Intervention Visit: Worksheet

CFHealthHub

Participant ID: **Date:**
Interventionist name: **Time:**

Consent visit Motivation rating (from COM-BBQ): ____
Consent visit Confidence rating (from COM-BBQ):____

1. I have turned on the Dictaphone ☐
2. Are there prescription changes needed Yes ☐ No ☐
   These are:

3. Complete the online consent form with the participant ☐
4. Briefly Introduce CFHealthHub

   Check My progress ☐ Settings ☐
   CFHH App for smartphones ☐
   Navigation bar (top right) ☐

   Encourage participant to navigate

   Personalisation settings:
   - Wallpaper explained ☐
   - Notifications ☐
   - Rewards messages ☐
   - Data sharing allowed ☐

5. Ask participant to navigate to My Toolkit and discuss ☐

   Say: *This is tailored content, specifically designed for you based on your prescription and the answers that you gave to the questionnaire*

   Show:
   My Treatment modules ☐
   Treatment video(s) ☐

6. Discuss Motivation rating ☐
   (see top of page 1 of worksheet)

   Say: *When you completed the questionnaires about the study you were asked to agree or disagree with the statement:*
   'I want to do all of my prescribed nebuliser treatment in the next 4 weeks'*. (see motivation rating above)*
   Why did you want to do your treatment that much?

   Key reasons for motivation given were:

   Reflect and reinforce reasons ☐

   Say: *So you want to do your treatment because.....*

   You're absolutely right doing your treatment will....

7. Show adherence graphs or charts, orientation to display and functions. ☐

Graphs:

Default ☐ Date slider ☐

Target line ☐ Traffic light colour scheme ☐

Hover for times ☐

Tables:

Weekly ☐ Absolute values ☐

Traffic light colour scheme ☐

Any treatment is a success: focus on the positives

8. Review adherence and focus on successes Yes ☐ N/A☐

Say: *What helped you to take your treatment?* **or:** *What was different about this day when you took your treatment*

Key factors resulting in success were:

9. Ask about their nebuliser treatment on one (or two) day(s) last week ☐

Say: *Tell me about your treatments on [Tuesday] - what did you take, when and in what order?*

Identify which treatments are being missed:

Look for a time when less treatment than normal was taken

10. Ask about factors affecting non-adherence Yes ☐ N/A☐

Say: *What got in the way of you taking your treatment?* **or:** *What was challenging this day when you did not take [all of] your treatment*

Key factors affecting non-adherence were:

11. Is motivation too low to continue? No ☐**Go to part 12** Yes ☐ **because:**

No/low intention to take treatment ☐ Identified from beliefs ☐

Shuts down/not bothered ☐ Possible depression referred to PI ☐ __/__/2017

Other:

Select one of these reasons and **Go to part 15**

12. a) Support participant to make action plan(s).

The participant should type their own plan(s) into the Action planning tool

Yes ☐	What is the cue (*if*) in the *if then* plan?	
No ☐	Because:	

b) Support participant to set personal target goal Yes ☐

Agreed goal is [ ] %

Ideal no. of treatments =
Planned no. of treatments =
Planned treatments/Ideal treatments =
Multiply by 100 =

Which drugs do these planned treatments relate to:

Drug	Ideal number of treatments	Planned number of treatments

The participant should type their own coping plan(s) into the tool

13. Identify and discuss key anticipated barriers in meeting goal and identify solutions Yes ☐ N/A☐

Problem/Barrier	Solution
1.	
2.	
3.	

14. Discuss confidence **(goal set)** Yes ☐

Say: *Now that we have set your goal at x and come up with some solutions to your problems let's rate how confident you feel that you can achieve that goal?*

I am confident that I can achieve my target goal in the next 4 weeks

Strongly disagree						Strongly agree
1	2	3	4	5	6	7

Remind about times of good adherence ☐
Remind about tools to help ☐
Go to part 16

> **Say:** *Remember you did manage to do all of your treatment when....* **or** *You've made a plan now that could help with that problem*

15. Discuss confidence **(no goal set)** Yes ☐

Focus on relationship building

> *You need to understand the motivational barriers to adherence. So ask open questions, listen carefully, and reflect back what is said to make sure that you understand*

> **Say:** *When you completed the questionnaires about the study you were asked to agree or disagree with the statement: **'I am confident that I can do all of my prescribed nebuliser treatment in next 4 weeks'.** You said (see rating at top of worksheet sheet)*
>
> *Why were you that confident?*
>
> *What might help you to feel more confident?*

Did you identify additional modules that should be added to ***My Toolkit***?
Yes ☐
No ☐

16. Are there any suitable videos to add to the toolkit at this time?

Yes ☐ No ☐

> *Choose videos that best match the characteristics of the participant e.g. gender, age, occupation, barriers encountered. (optional)*

17. Review the session Yes ☐

Remind how to log in ☐
Remind how to access ***My Toolkit*** ☐
Ask if participants have any questions ☐
Remind how to access adherence charts ☐
Remind who to contact if problems ☐

> **Say:** *The most important thing is that we begin to learn how to help you with your nebuliser treatments. If this/these plans don't work, then don't worry. If that's the case then we'll work to find other plans and solutions to suit you when we meet next time.*

18. Make/confirm appointment for next session Yes ☐

Date and time:<br>Location/Mode	
